# BMP-SMAD1/5 Signaling Regulates Retinal Vascular Development

**DOI:** 10.3390/biom10030488

**Published:** 2020-03-23

**Authors:** Andreas Benn, Florian Alonso, Jo Mangelschots, Elisabeth Génot, Marleen Lox, An Zwijsen

**Affiliations:** 1 Center for Molecular and Vascular Biology, Department of Cardiovascular Sciences, KU Leuven, 3000 Leuven, Belgium; 2 VIB-KU Leuven Center for Brain & Disease Research, KU Leuven, 3000 Leuven, Belgium; 3 Centre de Recherche Cardio-Thoracique de Bordeaux (INSERM U0145), Université de Bordeaux, 33607 Bordeaux, France

**Keywords:** arteriovenous malformations, BMP signaling, intussusceptive angiogenesis, retina development, SMAD1/5, sprouting angiogenesis, vessel regression

## Abstract

Vascular development is an orchestrated process of vessel formation from pre-existing vessels via sprouting and intussusceptive angiogenesis as well as vascular remodeling to generate the mature vasculature. Bone morphogenetic protein (BMP) signaling via intracellular SMAD1 and SMAD5 effectors regulates sprouting angiogenesis in the early mouse embryo, but its role in other processes of vascular development and in other vascular beds remains incompletely understood. Here, we investigate the function of SMAD1/5 during early postnatal retinal vascular development using inducible, endothelium-specific deletion of *Smad1* and *Smad5*. We observe the formation of arterial-venous malformations in areas with high blood flow, and fewer and less functional tip cells at the angiogenic front. The vascular plexus region is remarkably hyperdense and this is associated with reduced vessel regression and aberrant vascular loop formation. Taken together, our results highlight important functions of SMAD1/5 during vessel formation and remodeling in the early postnatal retina.

## 1. Introduction

Bone morphogenetic protein (BMP) signaling is a major regulator of blood vessel formation and maturation. Several studies have demonstrated distinct roles of BMP ligands, receptors, coreceptors and intracellular effectors in many steps of the angiogenic process [1,2,3,4,5,6]. Likewise, many BMP signaling components, including BMP9, activin-like kinase 1 (ALK1), BMP receptor type II (BMPRII), endoglin (ENG) and common-mediator SMAD4, have been linked – when mutated in their respective encoding genes – to human vascular diseases, including pulmonary arterial hypertension (PAH) and hereditary hemorrhagic telangiectasia (HHT) [3]. Interestingly, however, the principal intracellular effectors of these BMP signaling components, SMAD1 and SMAD5, have so far not been associated with the development of vascular diseases, prompting further investigations into their role in vascular development and function.

We previously demonstrated that a crosstalk between the intracellular BMP effectors SMAD1 and SMAD5 (in here: SMAD1/5) and Notch signaling is required for proper sprouting angiogenesis during mouse embryonic development [7]. After the initial formation of blood vessels via vasculogenesis, vessels form from pre-existing ones via sprouting or intussusceptive angiogenesis. During sprouting angiogenesis, tip cells form filopodia and start to migrate in the direction of a growth factor gradient, while trailing stalk cells proliferate and maintain connectivity to the parental vessel. The sprout elongates dynamically and subsequently fuses with another vessel to generate a perfused vessel [8]. During intussusceptive angiogenesis, transluminal pillars are formed by an invagination into the vascular lumen that ultimately splits the parental vessel into two new vessels [9]. Subsequent to the formation of the early network via sprouting and intussusceptive angiogenesis, the vasculature undergoes remodeling processes, such as vessel regression, to generate the mature vasculature [10].

The developing retinal vasculature is a well-acknowledged mammalian model system to study sprouting angiogenesis as it allows visualizing different steps of the angiogenic process simultaneously. The retinal vasculature starts to develop at postnatal (P) day 0 and expands radially from the optic nerve. In the following days, the vasculature expands via sprouting and intussusceptive angiogenesis, and is remodeled into the mature vascular tree [11,12]. In this study, we investigated the effect of inducible, endothelium-specific deletion of *Smad1*/*5* on early postnatal retinal angiogenesis. We show that conditional deletion of *Smad1/5* results in direct connections between arteries and veins (arteriovenous malformations, AVMs), fewer and less functional tip cells as well as a hyperdense vascular plexus. We further highlight a role of SMAD1/5 during vessel regression and a potential involvement in intussusceptive angiogenesis. In sum, our data provide a detailed view of the consequences of endothelium SMAD1/5 loss on retinal angiogenesis and vascular remodeling that sheds more light on their function in the endothelium and allows for a direct comparison with other studies performed in the postnatal mouse retina.

## 2. Materials and Methods

### 2.1. Animal Experiments

Homozygous mice for *Smad1/Smad5* floxed alleles (*Smad1^fl/fl^;Smad5^fl/fl^*) [7] were bred with transgenic mice expressing tamoxifen-inducible Cre in the endothelium (*Cdh5-CreERT2^tg/0^*) [13]. *Smad1^fl/fl^;Smad5^fl/fl^* dams were mated with resulting *Cdh5-CreERT2^tg/0^;Smad1^fl/+^;Smad5^fl/+^* mice to obtain *Cdh5-CreERT2^tg/0^;Smad1^fl/fl^;Smad5^fl/fl^*. Recombination events in the endothelium were visualized using a conditional Cre-reporter strain (RCE) with a CMV-IE enhancer/chicken beta-actin/rabbit beta-globin hybrid promotor (CAG)-boosted expression of enhanced green fluorescent protein (EGFP) in homozygous *RCE^+/+^;Smad1^fl/fl^;Smad5^fl/fl^* reporter dams [7]. Pups were injected intraperitoneally with tamoxifen (500 µg) at P2 and P3, and sacrificed at P4 or P6. Genotyping of recombined alleles was done as previously described [7]. For perfusion experiments, pups were anesthetized by placing them on ice for 10 min, injected retro-orbitally with fluorescein isothiocyanate FITC-coupled lectin (FL-1171; Vector Laboratories) and lectin was allowed to circulate 5 min before sacrifice. All experiments were conducted using littermate controls. The Ethical Committee of KU Leuven approval all animal procedures (P039/2017).

### 2.2. Reagents and Antibodies

All reagents and antibodies were purchased from Sigma-Aldrich (Overijse, Belgium), unless stated otherwise.

For immunofluorescence analysis, the following antibodies/reagents were used: biotinylated Griffonia simplicifolia lectin I (isolectin B4) (L2140; 1:40); mouse anti-alpha-smooth muscle actin-Cy3 (C6198; 1:200); goat anti-ephrin type B receptor 4 (E4779; 1:100); rabbit anti-phospho(p)SMAD1/5/9 (#13820, 1:100, Cell Signaling Technology; Bioké, Leiden, The Netherlands); rabbit anti-erythroblast transformation-specific related gene (ERG, ab110639, 1:200, Abcam, Cambridge, United Kingdom); rabbit anti-collagen IV (ab6586; 1:100, Abcam, Cambridge, United Kingdom); rat anti-endomucin (sc-65495; 1:100; Santa Cruz; Bio-Connect B.V., Huissen, The Netherlands). It is of note that the SMAD8 protein is encoded by the *Smad9* gene (which is used in the naming of the #13820 antibody). Appropriate streptavidin conjugates or species-specific Alexa Fluor-coupled secondary antibodies (all 1:200) were purchased from Jackson Immunoresearch (Bio-Connect B.V., Huissen, The Netherlands).

### 2.3. Immunofluorescence Analysis and Quantification

Pups were sacrificed at P4 or P6 and whole eyes were isolated and prefixed in 4% paraformaldehyde (PFA) for 20 min at room temperature. Subsequently, retinas were dissected, fixed in 4% PFA for 2 h at 4 °C and further processed for immunofluorescence analysis as previously described [14]. For pSMAD1/5/8 staining, retinas were incubated with the primary antibody for 72 h at 4 °C. Confocal microscopy of flat-mounted retinas was carried out on a Leica SP8 confocal microscope. Analysis and quantification of at least two images per retina were done in a blinded manner using ImageJ (National Institute of Health, Bethesda, Maryland, United States of America). Quantification of pSMAD1/5/8 was performed by manually counting the number of p-SMAD1/5/8 and IB4 double-positive endothelial cells (ECs) per field in the remodeling plexus, and the counts were normalized to the IB4-positive areas quantified using ImageJ software. Radial expansion measurements were performed on IB4-stained flat-mounted retinas by measuring the distance between the optic nerve center and the outmost tip cell. Quantification was done using the mean of eight individual measurements across the flat-mounted retina. Vessel inner diameter was measured on corresponding areas from three distinct points along the same vessel and the resulting means were used for further analysis. Fluorescence intensity was measured by marking a region of interest around individual arteries, veins or AVMs and measuring the signal intensity within the region of interest. Tip cell and filopodia measurements were performed using ERG/IB4 co-stained flat-mounted retinas. Tip cells were defined as the outermost single nuclear cells extending from the angiogenic front. Vascular density was determined on grayscale IB4-stained flat-mounted retinas by measuring the fluorescent area vs. the nonfluorescent area. Empty collagen sleeves and vascular loops were manually counted on Col IV/IB4 co-stained flat-mounted retinas. The mean vascular loop area was quantified on whole optical fields in the plexus region using ImageJ software.

Images were processed and compiled using Adobe Photoshop 2019 (Munich, Germany).

### 2.4. Statistical Analysis

Normal distribution was confirmed using the D’Agostino–Pearson normality test. A Welch’s t-test was used to determine statistical significance. *P-*value below 0.05 was considered statistically significant: * *p* < 0.05; ** *p* < 0.01; *** *p* < 0.001; **** *p* < 0.0001. Error bars represent standard deviation. Cre-negative littermates were always used as control group. GraphPad Prism 8.1.1 (San Diego, CA, United States of America) was used for statistical analysis of all quantitative data.

## 3. Results

### 3.1. Endothelium-Specific Deletion of Smad1/5 Results in AVM Formation

**Figure 1:**
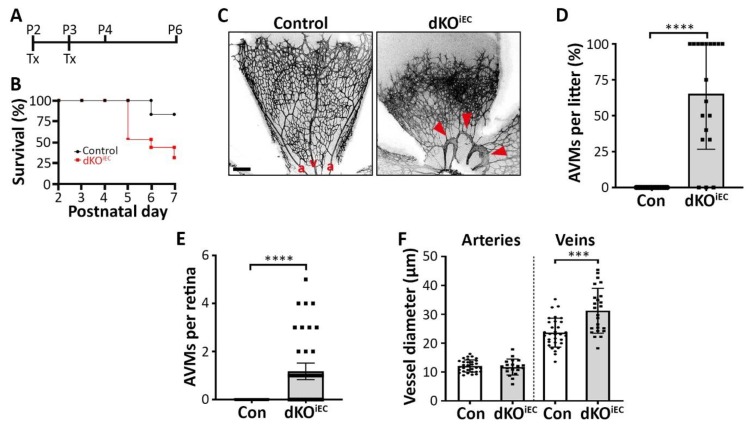
Endothelium-specific *Smad1/5* deletion results in arteriovenous malformation (AVM) formation in the retina. (**A**) Tamoxifen (Tx) treatment regime. Mice were injected with Tx at postnatal day 2 (P2) and P3, and analyzed at P4 or P6. (**B**) Survival rates of Tx-treated mice. Control *n* = 18. dKO^iEC^ *n* = 15. (**C**) Isolectin B4 (IB4) staining of Tx-treated control and double knockout mice (dKO^iEC^) retinas at P6. Red arrowheads indicate AVMs. a = artery. v = vein. Scale bar: 200 µm. (**D**) Quantification of AVMs in Tx-treated litters and (**E**) number of AVMs per retina at P6. 19 litters with control *n* = 82 and dKO^iEC^ *n* = 49. (**F**) Quantification of inner vessel diameter of arteries and veins in Tx-treated mice at P6. Control *n* = 31. dKO^iEC^ *n* = 19. *** *p* < 0.001; **** *p* < 0.0001.

We previously showed that a constitutive endothelium-specific double knockout of *Smad1/5* is embryonically lethal [7]; therefore, we generated tamoxifen-inducible, endothelium-specific *Smad1/5* double knockout mice (dKO^iEC^) to study their function during postnatal angiogenesis. Mice were treated with tamoxifen at P2 and P3 and subsequently analyzed at P4 or P6 (Figure 1A). Efficient Cre-mediated recombination in endothelial cells (ECs) was visualized using a reporter mouse strain (Figure S1A). Furthermore, we observed significantly fewer ECs with a pSMAD1/5/8 signal in dKO^iEC^ compared to control mice (Figure S1B,C). Overall, induced deletion of *Smad1/5* in the vasculature resulted in premature lethality compared to control mice (Figure 1B) and a 10% weight loss at P6 (Figure S1D). Therefore, we focused on the role of SMAD1/5 during early retinal vascular development. Injection of fluorescently-labeled lectin showed that retinas were healthy and fully perfused in dKO^iEC^ at P6 (Figure S1E,F). We observed AVMs at the first branch of major retinal arteries and veins in about 2/3 of dKO^iEC^ at P6 (Figure 1C,D). The malformations were already detected at P4, two days after induction of recombination, in about 15% of dKO^iEC^ (Figure S2A,B). The majority of dKO^iEC^ retinas exhibited one AVM and showed an increased inner vein diameter at P6 (Figure 1E,F), while no differences in vessel caliber were observed at P4 (Figure S2C).

Taken together, we show that endothelium-specific deletion of *Smad1/5* in postnatal mice results in AVM formation of major retinal arteries and veins.

### 3.2. Arterial- and Venous-Associated Markers Localize Normally in dKO^iEC^

**Figure 2:**
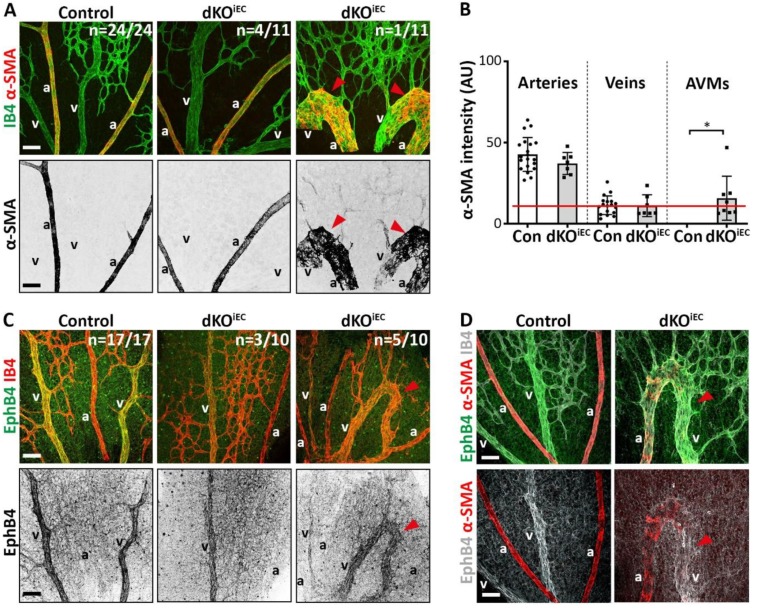
Arterial- and venous-associated markers localize normally in dKO^iEC^. (**A**) IB4 (green) and alpha smooth muscle actin (α-SMA, red) staining of Tx-treated control (*n* = 24) and dKO^iEC^ (*n* = 11; 4/11 without AVM formation; 6/11 with α-SMA-negative AVMs; 1/11 with α-SMA-positive AVM) retinas at P6. α-SMA grayscale images are shown for visual clarity. (**B**) Quantification of α-SMA signal intensity per vessel type (arteries, veins, AVMs) in Tx-treated mice at P6. The red line indicates background noise level. (**C**) Ephrin type b receptor 4 (EphB4, green) and IB4 (red) staining of Tx-treated control (*n* = 17) and dKO^iEC^ (*n* = 10; 3/10 without AVM formation; 2/10 with EphB4-negative AVMs; 5/10 with EphB4-positive AVMs) retinas at P6. EphB4 grayscale images are shown for visual clarity. (**D**) EphB4 (green/white), α-SMA (red) and IB4 (white) staining of Tx-treated control and dKO^iEC^ retinas at P6. Red arrowheads indicate AVMs. a = artery. v = vein. Scale bar: 50 µm. * *p* < 0.05.

It has been reported that endothelium-specific deletion of *Smad4* results in AVM formation and changes in arterial–venous identity in the retina of postnatal mice [5,15,16]. Considering the occurrence of AVMs in dKO^iEC^ pups, we tested whether changes in the localization of alpha smooth muscle actin (α-SMA), a marker of arterial-associated vascular smooth muscle cells (vSMCs), or venous-associated marker ephrin type b receptor 4 (EphB4) would occur in dKO^iEC^. About 36% (*n* = 4/11) of all analyzed dKO^iEC^ retinas showed no AVM formation and no changes in the exclusive arterial localization of α-SMA at P6 (Figure 2A, middle panel). While we observed AVM formation in about 64% of all analyzed dKO^iEC^ retinas (*n* = 7/11), only a minor fraction of them (*n* = 1/11) demonstrated α-SMA localization on an AVM (Figure 2A, right panel). Image quantification showed that α-SMA localization remained unaltered between dKO^iEC^ mice and control mice, and that α-SMA levels in AVMs were mostly below α-SMA background level (Figure 2B). Localization of EphB4 was unaffected in dKO^iEC^ mice showing no AVM formation (*n* = 3/10) (Figure 2C, middle panel) and was predominantly observed on AVMs (*n* = 5/10) (Figure 2C, right panel). The localization of arterial α-SMA and venous EphB4 identity markers remained fully intact outside of AVMs (Figure 2D).

Overall, our marker analysis suggests that arterial-venous specification proceeds normally in dKO^iEC^ and that AVMs are mainly of venous origin.

### 3.3. Endothelial SMAD1/5 Signaling Regulates Tip Cell Formation and Function During Retinal Sprouting Angiogenesis

**Figure 3:**
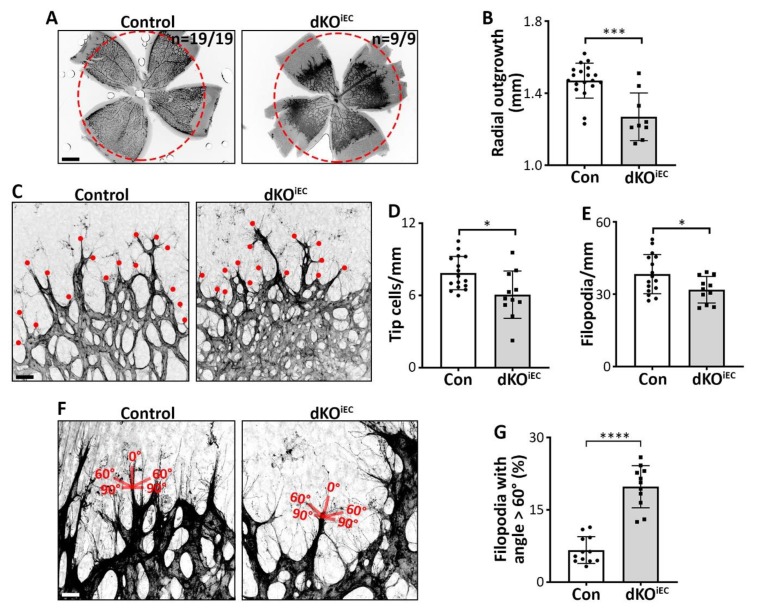
SMAD1/5 signaling regulates tip cell formation and function. (**A**) IB4 staining of Tx-treated control and dKO^iEC^ retinas at P6. The red dotted circle indicates radial outgrowth from control mice. Scale bar: 500 µm. (**B**) Quantification of radial outgrowth in Tx-treated mice at P6. Control *n* = 19. dKO^iEC^ *n* = 9. (**C**) IB4 staining of Tx-treated control and dKO^iEC^ retinas at P6. The red dots indicate tip cells. Scale bar: 25 µm. (**D**) Quantification of tip cells and (**E**) tip cell filopodia in Tx-treated mice at P6. Control *n* = 16. dKO^iEC^ *n* = 11. (**F**) IB4 staining of Tx-treated control and dKO^iEC^ at P6. The scheme represents the angle of filopodia from respective tip cells. Scale bar: 10 µm. (**G**) Quantification of filopodia with an angle > 60° respective to their tip cell in Tx-treated mice at P6. Control *n* = 12. dKO^iEC^ *n* = 11. * *p* < 0.05; *** *p* < 0.001; **** *p* < 0.0001.

Besides the formation of AVMs, we observed a hyperdense vascular network and a reduction in radial outgrowth with 100% penetrance in the retinal vasculature of dKO^iEC^ mice at P6 compared to control mice (Figure 3A,B). Since radial outgrowth is mainly driven by tip cell migration [17], we analyzed tip cell formation and function. Fewer tip cells and fewer filopodia were observed in dKO^iEC^ mice at P6 (Figure 3C–E). At P4, radial outgrowth was not yet significantly affected by the deletion (Figure S2A,D), and tip cell and filopodia numbers were comparable between dKO^iEC^ and control mice (Figure S2E,F). Tip cell filopodia sense growth factor gradients and, therefore, typically extend in an angle between 0° to 60° relative to the tip cell axis in the direction of the growth factor gradient [17]. We observed a higher percentage of tip cells with filopodial angles > 60° relative to the tip cell axis in dKO^iEC^ mice at P6 (Figure 3F,G). These findings reflect disturbed growth factor gradient sensing and/or directed migration of tip cells [18].

In sum, we report that *Smad1/5*-deficient ECs have fewer, less functional tip cells and show signs of migration defects.

### 3.4. dKO^iEC^ Mice Show Reduced Vessel Regression and Increased Vascular Loop Formation

**Figure 4:**
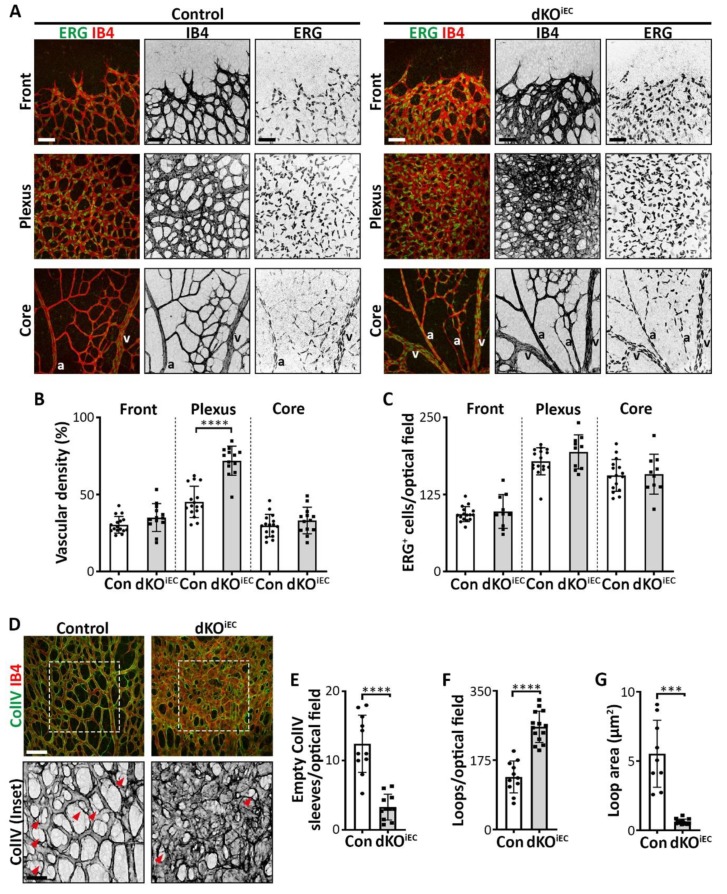
dKO^iEC^ mice show increased vascular density, reduced vessel regression and more abundant loop formation in the retinal plexus region. (**A**) ERG (green) and IB4 (red) staining of Tx-treated control and dKO^iEC^ retinas at P6. Retinal leaflets have been divided into three distinct regions: (i) the frontal region with migrating tip cells; (ii) the intermediate remodeling plexus region; and (iii) the mature core region around the optic nerve. Grayscale images of IB4 and ERG staining are shown for visual clarity. a = artery. v = vein. Scale bar: 50 µm. (**B**) Quantification of vascular density at the retinal vascular frontal, plexus and core regions of Tx-treated mice at P6. Control *n* = 16. dKO^iEC^ *n* = 13. (**C**) Quantification of ERG^+^ ECs at the retinal vascular frontal, plexus and core regions of Tx-treated mice at P6. Control *n* = 16. dKO^iEC^ *n* =10. (**D**) Collagen IV (ColIV, green) and IB4 (red) staining of Tx-treated control and dKO^iEC^ retinas at P6. ColIV grayscale images are shown for visual clarity. White dotted box highlights enlarged inset. Red arrows indicate empty ColIV sleeves. Scale bars: 50 µm (upper panel) and 25 µm (inset). (**E**) Quantification of empty ColIV sleeves in the retinal plexus region of Tx-treated mice at P6. Control *n* = 11. dKO^iEC^ = 11. (**F**,**G**) Quantification of the number of vascular loops and the mean vascular loop area in the retinal plexus region of Tx-treated mice at P6. Control *n* = 11. dKO^iEC^ = 14. *** *p* < 0.001; **** *p* < 0.0001.

Since we observed distinct effects in distinct regions of the retinal vascular network in dKO^iEC^ mice (Figure 3A), we segmented the retinal vasculature in three distinct regions that would allow for appropriate comparison with control mice: (i) the frontal region with migrating tip cells; (ii) the intermediate remodeling plexus region; and (iii) the mature core region around the optic nerve (Figure 4A). Vascular density was increased significantly in the plexus region of dKO^iEC^ mice at P6 (Figure 4B), while the density was increased in the frontal region at P4 compared to control mice (Figure S3A,B). Analysis of the endothelium-specific transcription factor ERG [14] demonstrated that total EC numbers were unaffected in dKO^iEC^ mice (Figure 4C), suggesting that a change in cell number did not contribute to the hyperdense plexus. We therefore hypothesized that vessel regression and/or intussusceptive angiogenesis could be deregulated in dKO^iEC^ mice. Vessel regression helps in establishing the mature vascular tree [10]. We observed fewer empty collagen IV sleeves in the plexus region of dKO^iEC^ mice at P6 (Figure 4D,E), indicating disturbed vessel regression. During blood vessel formation, sprouting and intussusceptive angiogenesis often occur in parallel, with sprouting angiogenesis contributing to the initial network, while remodeling vessels undergo intussusceptive angiogenesis to establish mature retinal vascular networks [12]. Retinal vascular loop formation has been associated with intussusceptive angiogenesis [19]. We observed significantly more vascular loops in dKO^iEC^ mice compared to control mice at P6 (Figure 4D,F). Loop area was significantly reduced upon the deletion of *Smad1/5* in ECs (Figure 4D,G).

Our data demonstrate that endothelium-specific deletion of *Smad1/5* results in a hyperdense vascular plexus network, due to decreased vessel regression and potentially increased intussusceptive angiogenesis during postnatal retinal vascular development.

## 4. Discussion

Our study highlights endothelial SMAD1/5 as regulators of distinct blood vessel formation phases in the early postnatal mouse retina and their deletion results in massive vascular defects. The presence of AVMs of the first branch point of arteries or veins in about two thirds of the retinas of dKO^iEC^ mice, suggested that AVM formation is not a fully penetrant phenotype in our model. Presence of AVM-like malformations were also found in embryos with an endothelium-specific deletion of *Smad1/5* [20]. To some extent, the retinal AVMs described here resemble the previously described AVMs of endothelium-specific *Smad4* knockout mice (*Smad4^iEC^*) [5]. Although AVMs in *Smad4^iEC^* mice were also formed in regions with high blood flow, the authors also report changes in the arterial-venous identity, specifically increased venous α-SMA coverage and arterial presence of the venous marker EphB4 in *Smad4^iEC^* mice. While AVMs are mostly of venous origin, we did not observe changes in the localization of arterial- and venous-associated markers in dKO^iEC^ mice. We also did not observe changes in the venous-exclusive presence of EphB4, suggesting that venous identity and EphB4 localization are unaffected by endothelium-specific deletion of *Smad1/5*. This finding is in contrast to a previous study describing a SMAD1/5 binding motif in an enhancer region of *EphB4* and a BMP-ALK3-SMAD1/5-dependent control of venous identity in mouse embryos and zebrafish [21]. We speculate that the observed differences in dKO^iEC^ mice compared to endothelium-specific deletion of *Alk1*, *Smad4* [5], *Alk3* [21] or *Eng* [3] are due to mechanistic differences in the resulting BMP-SMAD signal transduction in the respective mutants. Considering the role of these BMP signaling components in the pathogenesis of diseases such as HHT [2,3,5,22], an understanding of the underlying molecular mechanisms is paramount to the identification of novel therapeutic targets.

We also observed significantly reduced radial outgrowth, less functional tip cells and an increased density of the plexus region with full penetrance in all dKO^iEC^ mice during postnatal retinal angiogenesis. We previously showed that endothelium-specific deletion of *Smad1/5* results in more tip cell-like cells in the mouse hindbrain region at E9.5 due to a crosstalk between SMAD1/5 and Notch-dependent signaling [7]. While this seems to contradict our current data, results presented in this study are in line with a set of endothelium-specific BMP receptor knockouts in the early postnatal mouse retina. It has been reported that endothelium-specific knockouts of the BMP receptors *Alk1* (*Alk1^iEC^*), *Alk2* (*Alk2^iEC^*), *Alk3* (*Alk3^iEC^*) and *Bmpr2* (*Bmpr2^iEC^*) have very distinct outcomes on radial expansion of the retinal vasculature, tip cell formation and vascular density in transgenic mice at P5. While *Alk1^iEC^* mice exhibit no changes in radial outgrowth, but an increased number in tip cells and a higher vascular density [4,6], *Alk2^iEC^*, *Alk3^iEC^* and *Bmpr2^iEC^* mice demonstrate reduced radial outgrowth, tip cell number and vascular density [4]. Based on these findings, we speculate that tip cell formation and function are predominantly regulated by ALK2, ALK3 or BMPRII in a SMAD1/5-dependent manner in the early postnatal mouse retina. We hypothesize that loss of SMAD1/5 shifts endothelial BMP signaling towards SMAD2/3 signaling that has been shown to regulate vascular stability [23] and stalk cell competence [1], and can be activated by BMP receptors [24]. This shift could cause an imbalance in tip and stalk cell formation as observed here and elsewhere [4,6,7,18]. These findings also highlight the delicate relationship between the involved BMP receptors and coreceptors, their resulting heteromeric complexes, the activated downstream effectors and the resulting outcomes on sprouting angiogenesis. We previously identified that distinct BMP signaling modes depend on BMP receptor expression, which in turn is dependent on the vascular bed [25]. Thus, a differential expression of BMP signaling components likely explains the observed differences between endothelium-specific deletion of *Smad1/5* in different vascular beds.

Finally, we observed an increased vascular density in the plexus region of dKO^iEC^ mice. Considering the striking morphology of the dense plexus, we speculated that this hyperdensity could result from either a change in total EC numbers or EC size, vessel regression or intussusceptive angiogenesis. While EC numbers were comparable between dKO^iEC^ and control mice, we observed fewer empty collagen IV sleeves and more vascular loops that were indicative of reduced vessel regression and intussusceptive angiogenesis, respectively, in the plexus region. Although both mechanisms regulate vascular maturation, in-depth molecular details remain incompletely understood. Vessel regression, EC migration and intussusceptive angiogenesis are driven by blood flow but they respond to distinct blood flow profiles [10,12,26]. We observed significant hyperdensity in the plexus region, which is exposed to lower blood flow compared to the core region around the optic nerve [27]. Since BMP-SMAD1/5 signaling has repeatedly been shown to be sensitive to shear stress [27,28,29,30], we assume that SMAD1/5-dependent signaling controls vessel regression and intussusceptive angiogenesis in a blood flow-dependent manner.

Our study provides an insight into the role of SMAD1/5 during blood vessel formation in the postnatal mouse retina. We show that in dKO^iEC^ mice, AVMs form in areas of high blood flow, while reduced vessel regression and increased loop formation can occur in areas of lower blood flow. Besides, deletion of *Smad1/5* results in fewer and less functional tip cells. Collectively, these data highlight distinct roles of SMAD1/5-dependent signaling in the orchestrated process of blood vessel formation, ranging from tip cell migration to vessel remodeling. Future studies should address the specific molecular mechanisms of SMAD1/5-dependent signaling during distinct vascular development processes. Understanding these processes may lead to the identification of novel therapeutic targets for BMP-SMAD1/5-dependent vascular diseases, such as HHT.

## Supplementary Materials

The following are available online at https://www.mdpi.com/2218-273X/10/3/488/s1, Figure S1: Efficient recombination and perfusion in Tamoxifen-treated endothelial-specific Smad1/5 knockout mice at P6; Figure S2: Early postnatal stage deletion of Smad1/5 in endothelial cells results induces vascular malformations at P4; Figure S3: dKOiEC mice show increased vascular density in the front region at P4.
