# Supplementary material for: BMP-SMAD1/5 Signaling Regulates Retinal Vascular Development
Source: Biomolecules. 2020 Mar 23;10(3):488. doi: 10.3390/biom10030488 (PMC7175193; doi:10.3390/biom10030488)
Supplement: Supplementary file 1 [file biomolecules-10-00488-s001.pdf]

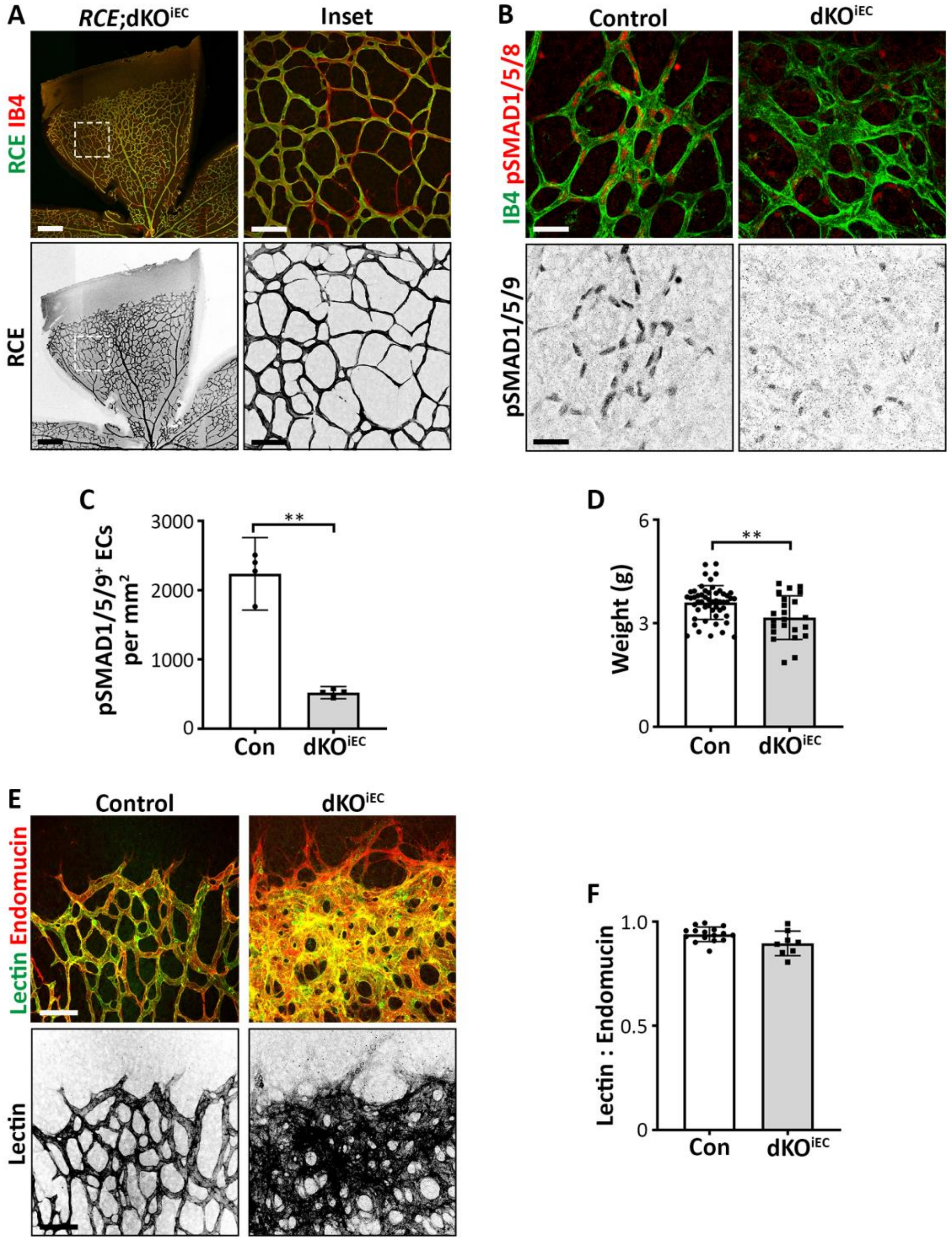

Supplementary Figure S1: Efficient recombination and perfusion in Tamoxifen-treated endothelial-specific *Smad1/5* knockout mice at P6.

(A) Isolectin B4 (IB4, red) staining of retinas from tamoxifen (Tx)-treated *RCE*;dKO^{iEC} mice expressing R26R CAG-boosted EGFP (RCE, green) upon Cre-mediated recombination at P6. Grayscale images of RCE are shown for enhanced visual clarity. White dotted box highlights

enlarged inset. Scale bars: 200 µm (left) and 50 µm (inset). (B) Phospho(p)-SMAD1/5/8 (red) and IB4 (green) staining of retinas from Tx-treated control and dKO^{iEC} retinas at P6. Grayscale images of pSMAD1/5/8 are shown for enhanced visual clarity. Scale bar: 50 µm. (C) Quantification of endothelial pSMAD1/5/8 intensity in Tx-treated mice at P6. Control n = 4. dKO^{iEC} n = 4. (D) Quantification of weight of Tx-treated mice at P6. Control n = 52. dKO^{iEC} n = 23. (E) Endomucin (red) staining of Tx-treated, Lectin-FITC-injected control and dKO^{iEC} retinas at P6. Grayscale images of Lectin-FITC are shown for enhanced visual clarity. Scale bar: 50 µm. (F) Quantification of Lectin vs Endomucin signal intensity in Tx-treated mice at P6. Control n = 16. dKO^{iEC} n = 8.

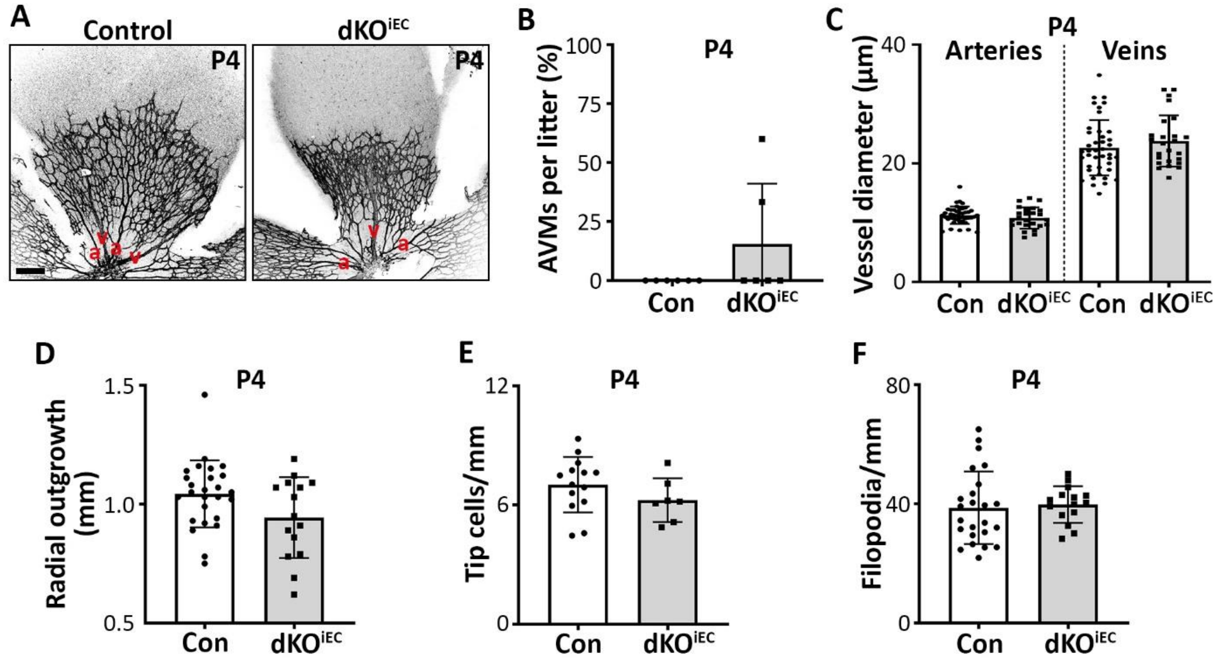

Supplementary Figure S2: Early postnatal stage deletion of *Smad1/5* in endothelial cells results induces vascular malformations at P4.

(A) IB4 staining of Tx-treated control and dKO^{iEC} retinas at P4. Scale bar: 200 µm. a = artery. v = vein. (B) Quantification of vascular malformations in Tx-treated litters at P4. 6 litters with Control n = 26 and dKO^{iEC} n = 16. (C) Quantification of inner vessel diameter of arteries and veins in Tx-treated mice at P4. Control n = 43. dKO^{iEC} n = 25. (D) Quantification of radial outgrowth in Tx-treated mice at P4. Control n = 24. dKO^{iEC} n = 15. (E) Quantification of tip cells in Tx-treated mice at P4. Control n = 14. dKO^{iEC} n = 7. (F) Quantification of tip cell filopodia in Tx-treated mice at P4. Control n = 24. dKO^{iEC} n = 15.

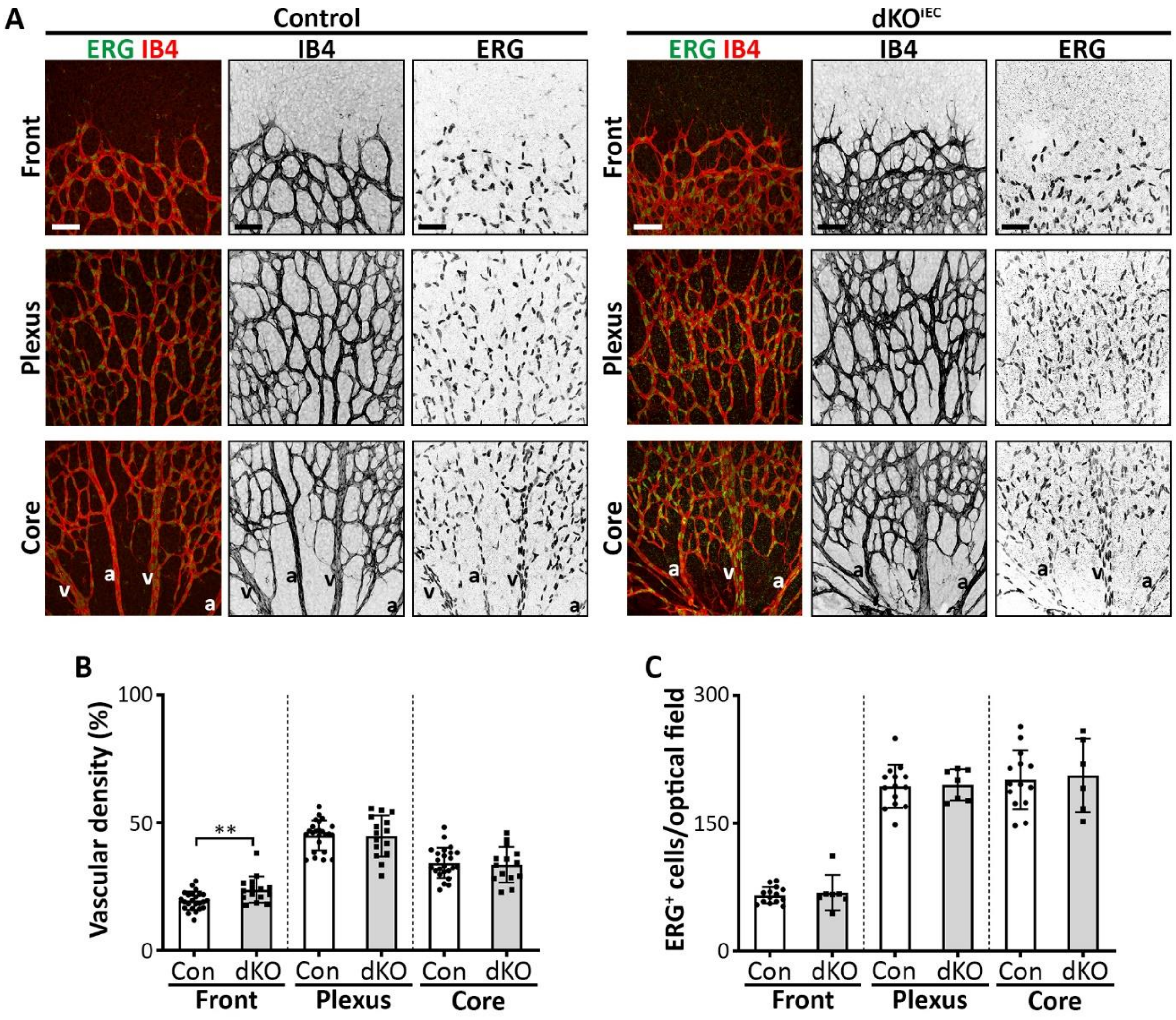

Supplementary Figure S3: dKO^{iEC} mice show increased vascular density in the front region at P4.

(A) ERG (green) and IB4 (red) staining of Tx-treated control and dKO^{iEC} retinas at P4. Retinal leaflets have been divided in three distinct regions: (i) the front region with migrating tip cells; (ii) the intermediate remodeling plexus region; and (iii) the mature core region around the optic nerve. Grayscale images of IB4 and ERG staining are shown for visual clarity. Scale bar: 50 µm. a = artery. v = vein. (B) Quantification of vascular density at the retinal vascular front, plexus and core region of Tx-treated mice at P4. Control n = 24. dKO^{iEC} n =15. (C) Quantification of ERG^{+} endothelial cells at the retinal vascular front, plexus and core region of Tx-treated mice at P4. Control n = 14. dKO^{iEC} n =7.
